# Family-led mid-upper arm circumference (FL-MUAC) approach and the screening of acute malnutrition in children aged 6 to 59 months in Africa: a scoping review

**DOI:** 10.11604/pamj.2024.49.38.36956

**Published:** 2024-10-11

**Authors:** Promise Rangarirai Majiwa, Prosper Chopera, Tonderayi Mathew Matsungo

**Affiliations:** 1 Department of Nutrition, Dietetics and Food Sciences, University of Zimbabwe, P.O Box MP 167, Mt Pleasant, Harare, Zimbabwe

**Keywords:** Wasting, growth monitoring, mid-upper arm circumference, infant and young child feeding, Africa

## Abstract

**Introduction:** family-led mid-upper arm circumference (FL-MUAC) is a community-based acute malnutrition screening approach that is centered on training the mother or caregiver to use colour-coded MUAC tapes to screen children for malnutrition. A scoping review was conducted to summarise available evidence and evaluate the use of the FL-MUAC approach in the screening for acute malnutrition in Africa. A systematic literature search was performed using electronic databases to identify relevant research documents investigating the FL-MUAC approach. The search sources included PubMed, Google Scholar, and institution websites such as the Emergency Nutrition Network (ENN) and The State of Acute Malnutrition. Documents were screened and assessed for eligibility and data was extracted from the eligible documents. Twelve documents were eligible for review based on the inclusion and exclusion criteria. Eight peer-reviewed articles and four operational documents were included in the scoping review. The results show that the FL-MUAC approach has been used by mothers and caregivers to monitor their children’s nutrition status in sixteen countries including Zimbabwe, Niger, Kenya, Chad, and Mali. In the sixteen countries where the approach has been implemented, there has been evidence of improved acute malnutrition screening coverage and increased frequency of screening, low hospitalization rate, and high cure rate of malnutrition cases attributed to the FL-MUAC approach. In conclusion, the FL-MUAC approach is still being piloted in most African countries. In addition, available evidence shows that FL-MUAC has the potential to be effective in early diagnosis and improved coverage for acute malnutrition. However, there is a need to integrate the FL-MUAC into healthcare systems and promote the standardization of monitoring and evaluation indicators.

## Introduction

Severe acute malnutrition (SAM) is a grave condition that significantly impacts the growth and development of children aged 0-59 months [1-5]. Prompt nutrition attention and medical support are imperative to mitigate the detrimental effects of SAM [6]. Early identification of affected children is paramount to ensure timely intervention through nutrition support programs [4,6,7].

Recognizing the urgency of early detection, the FL-MUAC approach has gained prominence as a community-based strategy for screening and identifying children at risk of acute malnutrition [8]. By empowering family members to measure a child's mid-upper arm circumference using a colour-coded tape, the FL-MUAC approach aims to enhance early detection and facilitate timely intervention [9]. This approach holds significant potential for improving community-led early detection of acute malnutrition cases, particularly in the context of the COVID-19 pandemic, where various lockdowns and restrictive measures have impeded access to healthcare services [10].

The integrated management of malnutrition model emphasizes community mobilization to identify malnourished children and provide appropriate care before severe complications arise [1]. Active screening, carried out by community health workers or volunteers, plays a pivotal role in this model. Mid-upper arm circumference (MUAC) measurements have emerged as a reliable diagnostic tool, enabling early identification of malnourished children and reducing child morbidity and mortality [11]. The COVID-19 pandemic has exacerbated the challenges of malnutrition, resulting in increased rates of acute malnutrition [10,12]. However, the coverage of services for screening, referral, and treatment has been hindered by restrictions imposed during national lockdowns, social distancing measures, and the inadequate availability of personal protective equipment (PPE) for healthcare workers [10]. Consequently, many children with acute malnutrition are diagnosed late, significantly increasing their risk of mortality.

To address these pressing issues, a scoping review was conducted to explore the use of the FL-MUAC approach as a screening tool for acute malnutrition in children aged 6 to 59 months in Africa. This review aims to summarize the available evidence evaluating the effectiveness of FL-MUAC in identifying malnutrition, its impact on clinical-level outcomes such as early treatment and time to recovery, the implementation modalities of FL-MUAC, and the existing gaps in its use for screening malnutrition. The following research questions were investigated: how effective is the FL-MUAC in identifying malnutrition? What is the impact of FL-MUAC on clinical-level outcomes such as early treatment and the time taken to recover? What are the implementation modalities of FL MUAC; and what are the gaps in the use of FL-MUAC in screening for malnutrition? By addressing these research questions, the review contributes valuable insights to policymakers, healthcare practitioners, and researchers, ultimately enhancing the screening and management of acute malnutrition in African contexts.

## Methods

**Identification of relevant and reliable studies:** a comprehensive search was conducted by three independent reviewers to identify relevant articles on the FL-MUAC approach. The search sources included PubMed, Google Scholar, institution websites such as the Emergency Nutrition Network (ENN) and The State of Acute Malnutrition; electronic bibliographic databases like Mendeley, and reference lists of relevant studies. The search strategy followed a population, interventions, control, and outcome (PICO) framework, as outlined in Table 1. The search terms and Boolean operators (AND, OR, and NOT) were utilized to create a comprehensive search syntax, incorporating keywords such as “family MUAC,” “MUAC AND child,” “caregiver OR parent,” “acute malnutrition,” and “community diagnosis OR community screening AND MUAC.” The search aimed to include articles published from 2015, when the program started, onwards.

**Table 1:** population, interventions, control, and outcome (PICO) framework

Population	Intervention	Comparison	Outcome
Caregivers with children aged 6-59 months who were screened for malnutrition using mid-upper arm circumference tapes in Africa	None required	None required	Screening frequency
	Any intervention at the community level with caregivers using mid-upper arm circumference tape for assessing malnutrition	compared with health worker usage	Admission rates
			Sensitivity
			Hospitalisation rates
			Knowledge on malnutrition
			Recovery rates
			Ready-to-use therapeutic food (RUTF) consumption rates

**Inclusion and exclusion criteria for studies:** to be included in the review, studies had to meet specific criteria. The study population should have comprised caregivers with children aged 6-59 months who were screened for malnutrition using MUAC tapes at the community or household level. Studies that utilized MUAC as a means of detecting or diagnosing malnutrition were eligible for inclusion. The research period for the studies considered should have fallen within the timeframe from 2015 onwards. The review included various types of documents, such as operational documents, observational studies, intervention studies, and reviews. Studies that did not involve caregiver use of MUAC or focused on infants younger than 6 months of age, as MUAC is not recommended for this age group, were excluded.

**Data collection methods:** data from the selected studies were collected by the three independent reviewers. The reviewers followed a standardized data extraction form or template in Word format to ensure consistency. Any discrepancies or disagreements in data extraction were resolved through discussion or by involving a third reviewer. For obtaining or confirming data from the study investigators, the reviewers attempted to contact them via email or other communication channels when additional information or clarification was required. This process ensured the accuracy and completeness of the extracted data.

**Collating and summarizing findings:** all articles and documents identified during the search were imported into a reference manager, and duplicates were removed. Relevant information from selected articles, including authors, country, participant characteristics, study design, key findings, data extraction, and collection were primarily collected manually by the reviewers using standardized forms or templates. The PRISMA guidelines for scoping reviews (PRISMA-ScR) were also used to present findings.

**Data synthesis and analysis:** a narrative synthesis approach was employed to summarize the findings from the included articles and documents. The key characteristics of the reviewed studies, such as study design, sample size, and geographical location, were tabulated in Table 2, and Table 2.1. The implementation strategies and outcomes of the FL-MUAC approach were analyzed qualitatively to identify common themes, trends, and challenges across the studies. “In this study, data were sought for the following outcome domains: caregiver compliance and satisfaction: This outcome domain focused on evaluating the level of caregiver compliance with FL-MUAC screening and their satisfaction with the approach. Data were sought for measures such as the percentage of caregivers correctly using the MUAC tape, caregiver-reported satisfaction scores, and qualitative feedback from caregivers.

**Table 2:** summary of findings from studies that assessed the family-led mid-upper arm circumference approach

Objective	Study design	Participants	Key findings	Location
To compare, under program conditions, mothers and CHWs in screening for severe acute malnutrition (SAM) by colour-banded mid-upper arm circumference (MUAC) tapes	pragmatic interventional, non-randomized efficacy study	12 893 mothers and caregivers	Mothers were not inferior to CHWs in detecting malnutrition	Niger
			There was regular MUAC screening by mothers	
			There was a higher median MUAC at admission and lower hospitalization rates at admission and during treatment.	
			Earlier detection of cases	
To test three simple MUAC classification devices to determine whether they improved the sensitivity of mothers/caregivers in detecting acute malnutrition	Prospective, non-randomized, partially blinded, clinical diagnostic trial	1 040 children assessed by mothers or caregivers	The sensitivity of mother/caregiver classifications was high for all devices (>93% for severe acute malnutrition (SAM), > 90% for global acute malnutrition (GAM).	Global
			Mother/caregiver sensitivity for SAM and GAM classification was higher using the MUAC insertion tape (100% sensitivity for SAM and 99% sensitivity for GAM) than using “Click-MUAC” devices.	
			The results of this study provide strong evidence to support the ability of mothers to perform sensitive and specific measurements of their child’s MUAC	
To determine whether a colour-banded MUAC strap would allow minimally trained mothers to screen their children for malnutrition, without locating the mid-point of the left upper arm by measurement, as currently recommended	A non-randomised non-blinded evaluation	103 mothers with children aged 6 to 59 months	Mothers had a sensitivity and specificity for classification of their child’s nutritional status of> 90% and > 80% respectively for global acute malnutrition and > 73% and > 98% for severe acute malnutrition.	Global
			The choice of the arm did not influence the classification results; a weighted Kappa of 0.88 for mothers and 0.91 for CHW represents almost perfect agreement.	
			There was no difference between the MUAC value between arms or the midpoint determined by measurement or by eye	
To assess the feasibility of shifting clinical surveillance to caregivers in the outpatient management of SAM	Proof of concept trial	128 caregivers of children with uncomplicated SAM	Knowledge of most clinical danger signs (e.g., convulsions, oedema, poor appetite, respiratory distress, and lethargy) was low (0-45%) before training but increased immediately after and was retained 28 days after training.	Burkina Faso
			Agreement between nurse’ caregiver mid-upper arm circumference colour classifications was 77% immediately after training and 80% after 28 days.	
To determine whether OptiMA (the strategy that trains mothers to use mid-upper arm circumference (MUAC) bracelets for screening and targets treatment to children with MUAC < 125 mm or oedema with one therapeutic food at a gradually reduced dose) conforms to SPHERE standards (recovery rate > 75 %).	single-arm proof-of-concept trial	4958 caregivers with children aged 6 to 59 months who had MUAC < 123 or oedema	Global recovery was 86.3% and 70.5% for children admitted with MUAC < 115 mm or oedema	Kenya
			Recovery was positively associated with mothers trained to use MUAC before their child’s admission	
			RUTF ration for children admitted with admitted SAM was reduced by nearly half at 72.2 sachets per child treated	
			An average of 54.3 RUTF sachets/child were consumed by children admitted with MAM which is lower than the 60-90 ready-to-use supplementary food sachets/child typically planned for MAM programming	

**Table 2.1:** summary of findings from studies that assessed the family-led mid-upper arm circumference approach

Objective	Study design	Participants	Key findings	Location
To present a clear overview of the available evidence as well summarize the available evidence, showing what is known about the impacts, outcomes and implementation of this approach and to appraise what is still not known, or difficult to establish (i.e. to identify critical weaknesses in the evidence and practice).	Rapid review	Children aged 6 to 59 months and caregivers	Based on operational findings and peer-reviewed studies, it is clear that caregivers can correctly take mid-upper arm circumference (MUAC) measurements.	Global
			In settings with a low prevalence of oedema, the ability of caregivers to detect oedema seems to be lower and yet aligned with the “global ability” (CHWs, health workers) of detecting oedema in these settings.	
			The MUAC protocol (either arm and visual ascertainment of midpoint) used by some implementers performed as well as the standard protocol for MUAC measurement	
			There is little evidence on the fact that a Family-MUAC approach can improve the quality of treatment by reducing the time needed for it and by fasting recovery	
			Some implementers have demonstrated an increased frequency of screening when done by caregivers which then indicates an improved coverage of screening.	
			Comparing coverage between the Family-MUAC approach and another standard mechanism such as CHW screening can produce more striking evidence to support advocacy and scale-up of the approach.	
			There is limited evidence on the cost-effectiveness of the family-MUAC approach.	
To summarize the published and operational evidence describing (1) the use of MUAC by caregivers and CHWs in community settings for the detection and diagnosis of SAM, (2) the treatment of SAM by CHWs in community settings, and (3) health platforms where MUAC use and SAM management have been successfully integrated	Systematic review	Children aged 6 to 59 months and caregivers	Caregivers can use MUAC to detect SAM in their children with little apparent risk and many potential benefits to early case detection and coverage.	Global
			Caregiver-focused models for detecting and classifying SAM using MUAC have the potential for increasing coverage and detecting acute malnutrition earlier than standard MUAC protocols.	
			Further simplification of MUAC protocols may hold additional potential in increasing effective community/caregiver MUAC use in other settings	
			In recognizing the benefit of MUAC as a community tool, it is important to recognize that current MUAC criteria do not select for all high-risk children, including low weight-for-height children, and the optimal approach will vary across different contexts. More research is needed to identify different options to identify these high-risk children in the community and ensure successful diagnosis and treatment.	
			Screening of children by caregivers significantly reduces after training therefore there is a need to continue motivating caregivers to screen their children	
To summarise available information on the family-led MUAC approach	Narrative review	Children aged 6 to 59 months and caregivers	The caregivers are capable of accurately measuring their child’s MUAC.	Global
			However, operational experiences highlight that capacity may decline as time passes after the last training.	
			In terms of timing of detection, there are promising results in operational findings and peer-reviewed studies supporting earlier detection.	
			There is little evidence linking family MUAC to a shorter length of stay in the program and/or an impact on program performance indicators. However, a recent trial (Daures *et al*., [5]) indicates that children of caregivers who received MUAC training were more likely to recover which could be explained by better care-seeking behaviour resulting from such training.	
			It is difficult to assess the impact of the Family MUAC approach on coverage of treatment independently from other factors (e.g., distance from a health facility) and this could explain why the impact on coverage for this approach is still unclear.	
			The review indicates that family MUAC can lead to an improved coverage of screening.	

**Community acceptance:** community acceptance of the FL-MUAC approach was assessed based on the findings from the included studies. The level of enthusiasm and participation among community members, including caregivers, community leaders, and young girls, was examined. The attitudes of men towards the approach were also explored to understand their level of engagement and support. The studies provided insights into the community's perception of the approach and its contribution to addressing misunderstandings regarding inclusion and exclusion in malnutrition programs.

**Caregiver compliance and satisfaction:** this outcome domain focused on evaluating the level of caregiver compliance with FL-MUAC screening and their satisfaction with the approach. Data were sought for measures such as the percentage of caregivers correctly using the MUAC tape, caregiver-reported satisfaction scores, and qualitative feedback from caregivers.

**Nutritional status:** this outcome domain aimed to assess the impact of the FL-MUAC approach on the nutritional status of children aged 6-59 months. Data were sought for measures such as weight-for-height z-score (WHZ), mid-upper arm circumference (MUAC), and prevalence of acute malnutrition.

**Effectiveness of the family-led mid-upper arm circumference approach:** the effectiveness of the FL-MUAC approach was assessed using various indicators derived from the reviewed studies. These indicators included the number of caregivers capable of measuring MUAC, the percentage of mothers who screened their children using the MUAC tape, the functionality of MUAC bracelets, correct utilization of the approach, MUAC utilization frequency in the last four weeks, and the sustainability of the approach. The findings from different studies were compared to determine the overall effectiveness of the FL-MUAC approach in screening for malnutrition.

## Results

**Figure 1:**
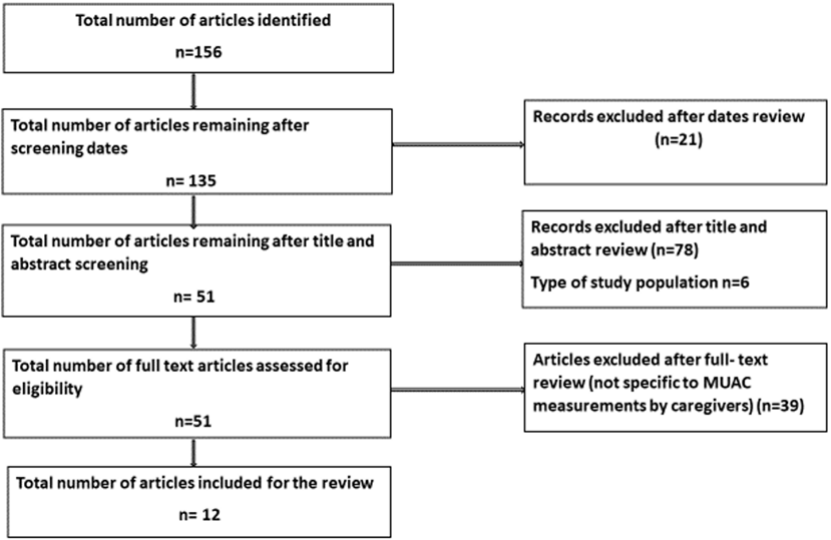
flow chart of the studies identification and selection process

**Screening and reviewed articles:** a total of 156 articles and documents were screened and twelve records were identified and selected for the review. A total of 123 articles were excluded due to different scopes (for example they investigated the efficacy of MUAC as a screening tool and use of MUAC tool by community health workers) as shown in Figure 1.

**Characteristics of reviewed articles:** a total of 12 resource documents were included; four were operational documents and eight were research articles. A summary of the studies is presented in Table 2, Table 2.1. All studies were conducted in rural setups with caregivers being trained to screen their children´s nutrition status using MUAC tapes.

**Implementation:** there is no standardized implementation approach to the strategy nor monitoring and evaluation strategy (Table 3). Family-led mid-upper arm circumference implementation approaches vary across organizations and countries. Action Against Hunger (ACF), ALIMA, and GOAL were major implementers of FL-MUAC [12]. Action against hunger piloted the approach in India, Mauritania, and Kenya whilst World Vision piloted it in Mauritania and ALIMA in Niger [12-14]. Other countries such as Kenya, Senegal, Zimbabwe, and Burkina Faso have also implemented the approach [1,2]. The selection of entry points for implementation depended on existing community mechanisms and available resources. Family-led mid-upper arm circumference could target various groups, including mothers with children aged 6 to 59 months, mothers with children suffering from severe acute malnutrition (SAM), women of reproductive age, pregnant mothers, fathers, and other family members. However, there was a lack of standardized implementation approaches, training standards, and monitoring and evaluation strategies [12]. The training was not uniform across the countries and implementing organizations because of the different implementing ways as well as the different community platforms that exist. Minimum training standards that encompass desired outcomes, cost-effectiveness, messaging, and data collection are needed to ensure uniformity and effectiveness.

**Table 3:** family-led mid-upper arm circumference implementing approach used in selected countries

Organization	Countries	Implementation approach
Action against hunger (ACF)	India	Contextual analysis
	Mauritania	Identifying activities for FL-MUAC coverage of the intervention: whether individual or group training, languages, and time frame
	Niger	Budget pieces of training follow-up and supervision cost
	Kenya	Implementing FL-MUAC
		Monitoring
Save the children	Not mentioned	Design identification of entry points, targets, and trainers
		Training of trainers
		Distribution of MUAC tapes
		Training of caregivers
		Follow-up
World vision	Mauritania	Design
		Training of trainers
		Training mothers/caregivers
		Monitoring and evaluation

**Community acceptance:** in Niger and Mauritania, community leaders were enthusiastic for local women to learn the skill and young girls were also keen on learning the skill as well as participating [14,15]. As generally known that men have less health-seeking behavior as compared to women, men in Mauritania were seen to not be interested but wanted their women to learn [15]. The community in Niger was delighted to finally know how some children were included in malnutrition programs therefore all misunderstandings regarding inclusion and exclusion in malnutrition programs were cleared [14].

**Early treatment and hospitalizations: impact of family-led mid-upper arm circumference on clinical-level outcomes:** implementation of FL-MUAC has shown positive impacts on clinical-level outcomes. In Niger, early detection through FL-MUAC screenings resulted in prompt treatment for malnutrition cases [16,17]. Children identified through FL-MUAC in Mauritania were admitted to malnutrition programs at an early stage, leading to reduced hospital stays and decreased reliance on inpatient care [15]. From programmatic data which was also collected in Mauritania, the frequency of screenings increased when mothers took multiple MUAC measurements, enhancing the chances of early detection and treatment of malnutrition [15]. The same results were also noted in studies done in Niger, Kenya as well as India [9,14,18]. Furthermore, overall data from various implanting partners showed that FL-MUAC screenings conducted by trained mothers before seeking health facility services demonstrated the impact of FL-MUAC in promoting early detection and treatment [12].

**Frequency of screenings:** there are no articles available with evidence on the FL-MUAC approach increasing the frequency of screening but operational findings have suggested that the approach increases the frequency of screenings. The frequency of screenings increases as mothers take several MUAC measurements as compared with village health workers [12].

**Ability of caregivers to measure mid-upper arm circumference:** various studies have shown that mothers can correctly attribute the MUAC colour class for their children and can accurately classify their child using either arm, with the mid-point of the upper arm ascertained by eye [14,16-18]. However, the ability of mothers to detect oedema was seen to be less for mothers who lived in areas with a low prevalence of oedema [16]. Accuracy was seen to not be determined by the arm used [14]. The ability of a mother to screen their children for malnutrition was seen to decrease with time [14]. This could be due to a lack of constant practice.

**Effectiveness of the family-led mid-upper arm circumference approach:** studies have shown that FL-MUAC is effective in identifying malnutrition cases [8,12,14,16,18]. Trained mothers and caregivers were able to accurately attribute the MUAC colour class for their children, indicating the effectiveness of FL-MUAC in screening. Early identification of malnutrition cases through FL-MUAC screenings led to timely admission to malnutrition programs, facilitating early intervention [17]. The operational findings demonstrated that a significant percentage of trained mothers actively screened their children using MUAC, reinforcing the effectiveness of FL-MUAC in identifying malnutrition cases. Precisely, an average of 72.3% of mothers took at least one measurement per month [12]. According to a study done in Mauritania, 83% of trained mothers screened for MUAC and oedema before bringing their child to the health center [15]. The lowest recorded was 20% cited by Burkina Faso which was assessed 10-14 months after training and no follow-ups were conducted after training [12]. Therefore, it can be concluded that FL-MUAC is effective in screening for malnutrition but it is affected by a lack of support after training as the frequency of screenings is reduced with time.

Operational findings showed that an average of 44.4% of admitted malnutrition cases were referred by mothers and there was a slight increase in referrals done by CHWs [12,15]. This indicator is an underestimate of the true level of screenings as mothers go to CHWs for confirmation and the admissions are then recorded as CHWs- admissions. The approach is relatively recent and not yet integrated into health systems therefore the indicator is generally underreported. All (100%) of the trained mothers screened their children before coming to the facility, and 80% suspected were confirmed SAM cases therefore FL-MUAC proved to be effective in screening for malnutrition [15]. There was a slight increase (50.1% to 56.1%) in screening coverage, with a majority of mothers reported to be screening their children several times a month therefore FL-MUAC enables children to be screened for malnutrition on multiple occasions leading to early detection of malnutrition [12].

**Impact of family-led mid-upper arm circumference on clinical-level outcomes:** implementation of FL-MUAC has shown positive impacts on clinical-level outcomes. Early detection through FL-MUAC screenings resulted in prompt treatment for malnutrition cases [14]. Children identified through FL-MUAC were admitted to malnutrition programs at an early stage, leading to reduced hospital stays and decreased reliance on inpatient care. The frequency of screenings increased when mothers took multiple MUAC measurements, enhancing the chances of early detection and treatment of malnutrition [8]. Furthermore, FL-MUAC screenings conducted by trained mothers before seeking health facility services demonstrated the impact of FL-MUAC in promoting early detection and treatment.

## Discussion

This study was conducted with the aim of summarising available evidence evaluating the use of the FL-MUAC approach in the screening for acute malnutrition in African settings. Results showed that mothers were able to measure MUAC and correctly attribute the MUAC colour class for their children and implementation of the approach led to increased frequency of screening, and fewer hospitalizations of children as malnutrition is detected early [3,8,14,16,17]. It was observed from our review that most assessments were conducted soon after the training which gives bias on the ability of mothers performing the tasks over time. Assessment should be carried out months after the training as well as assess the frequency of screening done at home as this highlights the ability of caregivers to screen and monitor their children at home. Most studies focused on training caregivers and health workers without considering other community members such as family members, faith healers, and community leaders. These are important in strengthening the approach and ensuring that the program continues to be implemented even in the absence of the caregiver.

Accurate measurements are dependent on the MUAC tape being in good condition therefore there is a great need for emphasis on proper caring practices for the MUAC tape such that it will not be folded or lost. Integrating the family MUAC into existing health platforms and systems as well as other programs or existing activities such as malnutrition treatment programs, vaccination campaigns, care group activities, mother to mother support groups and cooking demonstrations will ensure that mothers keep getting refresher pieces of training. Programs could be designed to facilitate mothers monitoring their children once entered into CMAM program to reduce the number of repeat visits especially where there is a great distance to the treatment centre and this will ensure a decrease in defaulter rates in the acute malnutrition program.

The approach was seen to be sustainable and does not require monetary motivation however it does require a certain level of motivation [15]. The success of the program depends on health care workers as there is a need to ensure that the measurements are being taken as well as adequate sensitization and advocacy for local leaders. Therefore, there is a need for enthusiastic staff to assure successful implementation, monitoring as well as evaluation. The approach also benefits community health workers as they become less overburdened with health tasks if mothers screen children on their own [14,18]. However, there is a need for intense nutrition education to improve acceptability as well as assure health workers of their importance because some health workers feared losing their importance in the community thereby reducing the acceptability of the approach at the health centre level [14].

It was noted that capacity and frequency of measurements done by caregivers decrease over time, hence the need for refresher training [8,10,18,19]. Caregivers who live in areas where there are low prevalence of oedema, were seen to less likely detect oedema [14]. This was also seen in communities that have low prevalence of malnutrition the intake and interest in the FL-MUAC approach was less as compared to communities with high prevalence [12]. A few setbacks during implementation were noted. The major barriers include distance, lack of awareness of malnutrition programs as well as previous rejection which resulted in low uptake and lack of interest in some countries [9,10]. Mass sensitization from training and repeat opportunities for health education activities help address these coverage barriers. There is limited research which has been done on the FL-MUAC approach. There were no studies found which were done in Southern Africa, with a few published articles from a few African countries namely Kenya, Mali, Chad, and Niger. Among the possible explanations were, inconsistent and incomplete reporting of FL-MUAC from the communities, as well as not publishing any work done on the approach.

**Gaps in implementing the FL-MUAC approach:** several gaps were identified in the use of FL-MUAC for screening purposes. The absence of standardized implementation approaches and monitoring and evaluation strategies hindered the uniformity and effectiveness of FL-MUAC programs. Sustainability was a concern, as the frequency of screenings decreased over time, indicating the need for ongoing support and reinforcement. Additionally, underreporting of screening coverage and referrals by mothers was observed due to their seeking confirmation from community health workers. Integration of FL-MUAC into health systems is still in progress, and the underreporting issue needs to be addressed to ensure accurate data collection and analysis [9,10].

Screening can stop or be reduced when partners are absent or when communities are not given support after training as cited by the case study done in India where the frequency of screening was 30% and in Mauritania at 20% [12]. There is a need to integrate the FL-MUAC into health systems such that caregivers can sustain the frequency of screenings. There is also a need for standardised monitoring and evaluation strategies that can gather data, conduct regular assessments of caregiver ability and make comparisons in terms of coverage with another screening mechanism. Integration of refresher training in the health systems platform is essential as both the ability of the caregiver and MUAC utilization decreases with time.

**Strengths of this scoping review:** the review applied a rigorous search strategy that retrieved articles to answer the research question and objectives. The topic focused on the effectiveness of the FL-MUAC approach in screening for malnutrition a little researched area hence our results add to the body of knowledge useful for upscaling this approach. Several electronic databases were used as primary sources. Multiple keywords were used to get the targeted relevant studies. However, some relevant studies for example, in foreign language (non-English) might have been omitted thereby being a limitation to the study.

## Conclusion

In countries where the approach was implemented, there was evidence of effectiveness of the FL-MUAC approach in screening for malnutrition, low hospitalization rate, and high cure rate of malnutrition cases. It can therefore be concluded that caregivers can perform sensitive and specific classifications of their child´s nutrition status using MUAC tapes and they are capable of diagnosing malnutrition in their children which could lead to early detection at a community level. Mothers were not inferior to community health workers in screening for malnutrition and the family-led MUAC approach has increased malnutrition awareness in caregivers as well as in the community.

Family-led mid-upper arm circumference has resulted in early treatment as children are identified early through frequent screenings which leads to early admission into malnutrition programs resulting in shorter hospital stays there for fewer children who need inpatient care. Family-led mid-upper arm circumference has been shown to lead to improved coverage of screenings as mothers seem to take several MUAC measurements per month. However, there is a need for intense nutrition education to improve acceptability as well as refresher training as the capacity and frequency of measurements done by caregivers decreases over time. Barriers such as distance and lack of awareness were noted during implementation which resulted in low uptake and lack of interest in some communities.

Limited research has been done on the FL-MUAC approach, thus future studies that evaluate the coverage and effectiveness of the FL-MUAC approach in different contexts are recommended. There is also a need to integrate the FL-MUAC approach which has been piloted by partner organizations into national health systems to ensure that mothers regularly obtain refresher training to ensure its effectiveness and sustainability, especially in times of pandemics like COVID-19. Furthermore, there is also a need for universally standardized training, implementation approach, and monitoring and evaluation criteria to enable scale-up and cross-country comparisons.

The current evidence shows the need for more studies to be done on the FL-MUAC approach especially in countries where there were no published research articles. More studies will help enrich knowledge on the implementation and impacts of FL-MUAC in different contexts. The insights gained from this study will encourage practitioners to implement the FL-MUAC as well as publish their results [8].

### What is known about this topic

- Using MUAC and weight for height (WHZ) for diagnosis, provides similar prevalence rates of acute malnutrition;
- Mid-upper arm circumference is an important tool for screening for acute malnutrition in emergency and low-income settings;
- Mid-upper arm circumference provides an accurate diagnosis and facilitates early identification of malnourished children thus reducing child-related morbidity and mortality.

### What this study adds

- This paper provides a summary of evidence on the effectiveness of the FL-MUAC approach in the screening for acute malnutrition;
- Adds to existing literature that shows the benefits of FL-MUAC to early detection of acute malnutrition in low-income and crises;
- The available evidence guides the program managers and policymakers seeking to scale up FL-MUAC programs in African settings.
